# Adventitial Progenitor Cells of Human Great Saphenous Vein Enhance the Resolution of Venous Thrombosis via Neovascularization

**DOI:** 10.1155/2021/8816763

**Published:** 2021-02-23

**Authors:** Siying Ling, Zhen Ma, Yong Teng, Xuemei Jiang, Junning Cheng, Ruihao Li, Mingyi Zhang, Hailong Luo, Yikuan Chen

**Affiliations:** ^1^Department of Vascular Surgery, Second Affiliated Hospital, Chongqing Medical University, Chongqing, China; ^2^Department of Hematology, Second Affiliated Hospital, Chongqing Medical University, Chongqing, China

## Abstract

**Background:**

Vascular adventitia contains progenitor cells and is shown to participate in vascular remolding. Progenitor cells are recruited into the venous thrombi in mice to promote neovascularization. We hypothesized that the adventitial progenitor cells of human great saphenous vein (HGSV-AdPC) enhance the resolution of venous thrombosis via neovascularization.

**Methods:**

Human great saphenous vein (HGSV) was harvested from the patients with great saphenous vein varicose and sectioned for immunohistochemistry, or minced for progenitor cell primary culture, or placed in sodium dodecyl sulfate solution for decellularization. Human venous thrombi were collected from patients with great saphenous vein varicose and superficial thrombophlebitis. Infrarenal abdominal aorta of New Zealand white rabbits was replaced with interposing decellularized vessel, and the patency of the grafts was confirmed by ultrasonic examination. Animal venous thrombi in the left infrarenal vena cava of mice were produced with Prolene suture ligation and ophthalmic force clipping of this portion. After HGSVs were digested by collagenase, the CD34^+^CD117^+^ HGSV-AdPC were isolated on FACS system, labelled with CM-Dil, and transplanted into the adventitia of infrarenal vena cava of nude mice. The percentage of thrombus organization area to the thrombus area was calculated as the organization rate. The thrombus cell, endothelial cells, and macrophages in the thrombi were counted in sections. Cell smears and frozen sections of human saphenous veins and venous thrombi were labeled with Sca1, CD34, CD117, Flk1, CD31, and F4/80 antibodies. The CD34^+^CD117^+^ HGSV-AdPC were cultured in endothelial growth medium with vascular endothelial growth factor (VEGF) to induce endothelial cell differentiation and analyzed with real time-PCR, Western blotting, and tube formation assays.

**Results:**

Immunohistochemical staining showed that the CD34^+^CD117^+^ cells were located within the adventitia of HGSVs, and many CD34^+^ and CD117^+^ cells have emerged in the human venous thrombi. The number of progenitor cells within the marginal area of 7 days mice thrombi was shown to be Sca1^+^ ≈21%, CD34^+^ ≈12%, CD117^+^ ≈9%, and Flk1^+^ ≈5%. Many CD34^+^adventitial progenitor cells have migrated into the decellularized vessels. FACS showed that the number of CD34^+^CD117^+^ HGSV-AdPC in primary cultured cells as 1.2 ± 0.07%. After CD34^+^CD117^+^HGSV-AdPC were transplanted into the adventitia of nude mice vena cava with venous thrombi, the organization rate, nucleate cell count, endothelial cells, and macrophage cells of thrombi were shown to be significantly increased. The transplanted CD34^+^CD117^+^ HGSV-AdPC at the adventitia have crossed the vein wall, entered the venous thrombi, and differentiated into endothelial cells. The CD34^+^CD117^+^ HGSV-AdPC in the culture medium in the presence of VEGF-promoted gene and protein expression of endothelial cell markers *in vitro* and induced tube formation.

**Conclusions:**

HGSV-AdPC could cross the vein wall and migrate from the adventitia into the venous thrombi. Increased HGSV-AdPC in the adventitia has enhanced the resolution of venous thrombi via differentiating into endothelial cells of neovascularization.

## 1. Introduction

Venous thrombosis is a common disease, with an increased annual incidence in recent years that varies from 104 to 183 per 100,000 person years in Europe [1, 2]. Thrombolysis and anticoagulant therapy are the main treatment strategies for venous thrombosis but have limited effects on venous thrombi, causing a severe risk of hemorrhage. Currently, highly effective methods combined with contraindications to anticoagulation and thrombolysis, such as gastrointestinal activated ulcer or brain hemorrhage, are needed for patients with venous thrombosis. Researchers are paying more attention to explore the new treatment methods for early resolution of venous thrombosis, which assists in significantly reducing the venous valve fibrosis as well as the incidence of postthrombus syndrome [3, 4].

The treatment for venous thrombosis mainly depends on its slow and natural resolution process [5, 6]. The natural resolution mechanism of venous thrombosis *in vivo* depends on thrombi organization, wherein the neutrophils, macrophages, and progenitor cells enter into the thrombus, and neovascularization appears within the body of the thrombus. Modarai et al. have found that bone marrow-derived endothelial progenitor cells are recruited into the thrombus of mice to promote neovascularization, but the cells were not found in the new vascular channels [7]. The progenitor cell mobilization with granulocyte colony-stimulating factor has enhanced the resolution of venous thrombi [5]. However, the derivation of progenitor cells still remains to be unclear.

The adventitial progenitor cells of vascular wall play an important role in the development and progression of vascular disease [8]. Campagnolo et al. have found that the adult human great saphenous vein (HGSV) contains perivascular progenitor cells with clonogenic and proangiogenic potential [9]. Hence, this study is aimed at exploring whether adventitial progenitor cells of human great saphenous vein (HGSV-AdPC) could enhance the resolution of venous thrombosis as well as investigate the possible underlying mechanism by neovascularization.

## 2. Materials and Methods

### 2.1. Collection of Vein and Thrombi from Humans

Human great saphenous veins (HGSV) were collected from great saphenous vein varicose patients who accepted great saphenous vein stripping at the Second Affiliated Hospital of Chongqing Medical University. Human venous thrombi were collected from patients with great saphenous vein varicose and superficial thrombophlebitis. This study was approved by the Institutional Review Board of the Chongqing Medical University, and informed consent was obtained from all patients.

### 2.2. Decellularized Vessel Preparation and Infrarenal Abdominal Aorta Transplantation

The procedure used for decellularized vessel was slightly modified to that described previously [10]. Briefly, HGSV was harvested from patients with great saphenous vein varicose and washed with phosphate-buffered saline (PBS) solution 6 times via puncturing at the distal end. The vein was cut at a length of 2 centimeters and placed in 0.75% sodium dodecyl sulfate (SDS) (Sigma, St. Louis, USA) solution on a 125 r/min shaker at 37°C for 8 hours. The vessels were then washed with PBS 5 times for a period of 10 minutes each on a shaker and then stored at 4°C in PBS with 1% heparin. Adult male New-Zealand white rabbits (weight, 2.0-2.5 kg, *n* = 5 per group) were anesthetized using 3% pentobarbital sodium (1 ml/kg) via the auricular vein. A midline laparotomy was performed to expose the infrarenal portion of the abdominal aorta to reach the confluence of the common iliac artery. Perivascular tissue was dissected by forceps, and tributaries were ligated. After heparinization at 150 IU/kg body weight, both the proximal and distal positions were cross-clamped. A 1.5-centimeter segment of infrarenal abdominal aorta was replaced with interposing decellularized vessel followed by end-to-end anastomosis with 7-0 Prolene sutures (Ethicon, Shanghai, China). The patency of the grafts was confirmed by ultrasonic examination (MyLab 90, Esaote, Italia). The animals had free access to water and standard laboratory rabbit chow, and no anticougulants were given after surgery. On postoperative days 7 and 14, the transplanted grafts were harvested for histologic analysis.

### 2.3. Adventitial Cell Primary Culture and CD34^+^CD117^+^ HGSV-AdPC Isolation

The procedure in this study was similar to that described previously [11]. The saphenous veins were flushed with PBS containing streptomycin-penicillin (100 U/ml), minced into fragments and digested by 0.1% type II collagenase (Sigma, USA) for 6-7 hours at 37°C in a water-bath, and then, the cell solution was filtered, centrifuged, and suspended. The suspended vascular wall cells were cultured in a septic bottle with 3 ml culture media (DMEM/F12, Lonza) containing 5% fetal bovine serum (Gibco), streptomycin-penicillin (100 U/ml, Hyclone), ITS (0.2%, Lonza), and LIF (0.1%, Sigma). The CD34^+^CD117^+^ HGSV-AdPC were isolated from passage 3 vascular wall cells on FACS system (BD Biosciences, USA). The target cells were used for culturing. To test the purity of HGSV-AdPC, the HGSV-AdPC (1 × 10^7^/ml) were washed with PBS and then blocked in PBS (10% BSA) for 20 min at room temperature, followed by incubation with mice anti-CD34-FITC and anti-CD117-APC (Abcam, USA) antibodies for 30 min at 4°C in the dark. The cells were further washed 3 times in PBS, resuspended in 500 *μ*l PBS, and observed under microscopy.

### 2.4. CD34^+^CD117^+^ HGSV-AdPC Labeled with CM-Dil

CM-Dil is a molecular probe that labels the living cells without affecting their activity. CM-Dil was dissolved with DMSO and diluted with PBS to make a concentration of 1 *μ*g/ml. CD34^+^CD117^+^ HGSV-AdPC were incubated first at 37°C for 5 minutes and then at 4°C for 15 minutes with CM-Dil. After labeling, the cells were washed with PBS twice and rinsed in fresh medium to remove excess fluorescent dye. The cells were resuspended in fresh medium and kept on ice for transplantation or photography under an inverted fluorescence microscope (Olympus, Japan).

### 2.5. Venous Thrombosis Model of Mice and Tissue Harvesting

All performed procedures were done according to the protocols approved by the Ethics Committee for Use and Care of Laboratory Animals of Chongqing Medical University. Venous thrombus in the left infrarenal vena cava of male C57BL/6 mice was produced by slightly modifying the previously described procedure [5, 7]. Male mice (weight, 20-25 g, 6-8weeks, *n* = 5 per group) were anesthetized, underwent midline laparotomy to expose the infrarenal vena cava to reach the confluence of the common iliac vein, and then isolated and separated from the adjacent abdominal aorta. The left infrarenal vena cava was ligated with 6-0 Prolene suture, and an ophthalmic force clipped this portion for 60 seconds on 2 occasions, with 30 seconds apart, and to induce endothelial damage. The intestines were replaced, and the abdominal wall was closed by the suture. At postoperative days 7 and 14, the mice were sacrificed by cervical dislocation. A laparotomy was performed to excise the infrarenal vena cava for histology from the ligature to the confluence of the common iliac veins.

### 2.6. CD34^+^CD117^+^ HGSV-AdPC Transplantation at the Adventitia of Infrarenal Vena Cava

Thrombi were induced as described above in the male nude mice. FACS purified CD34^+^CD117^+^ HGSV-AdPC passaged 3 generation. CM-Dil-labeled or CM-Dil-nonlabeled D34^+^CD117^+^ HGSV-AdPC were mixed with Matrigel Matrix (Catalogue reference:354248, Becton Dickinson, Bedford, MA, USA) at 4°C, and a final cell concentration of 10^6^ cells/ml was obtained. To test the effect of progenitor transplantation on resolution of venous thrombi, the mice in the treatment group underwent venous thrombi induction procedure, and 200 *μ*l HGSV-AdPC were seeded on the adventitia of infrarenal vena cava, while the control group mice underwent venous thrombi induction procedure and 200 *μ*l Matrigel Matrix suspension. To explore the migration of adventitial progenitor cells into the venous thrombi, 200 *μ*l labeled HGSV-AdPC were seeded in the adventitia of infrarenal vena cava of mice with venous thrombi.

### 2.7. Histology

The procedure used was similar to that described previously [5]. The HGSV, rabbit graft from infrarenal abdominal aorta, human venous thrombi, and mice venous thrombi were fixed in 4% paraformaldehyde for overnight and embedded in paraffin wax. The graft from the rabbit and thrombi from mice were stained with hematoxylin and eosin (HE) for histological evaluation. Transverse sections (5 *μ*m thick) were cut at 150 *μ*m intervals below the ligation throughout the length of the sample. Ten to 15 sections of each thrombus were obtained for analysis. Sections were viewed at ×40 magnification. Images (×400 magnification) of the whole section area were obtained and tiled to make a single composite with image analysis software (Image Pro Plus 6.0). The thrombus organization area and the thrombus area in each section were measured with the same software. Inflammatory cellular infiltration and fibrosis were considered to distinguish the organization area from the thrombus itself. The percentage of thrombus organization area to the thrombus area was calculated as the organization rate of each section. An average value of all sections of each animal was calculated as the organization rate of that animal. Sections were processed for immunohistochemistry for localization of CD34^+^, CD117^+^, CD31^+^, and F4/80^+^ cells using the antibodies. The antigen was retrieved at 650 W for 10 minutes in citrate buffer (pH, 6.0). A biotinylated secondary antibody was used to detect the binding of primary antibodies, followed by a streptavidin-peroxidase complex. The thrombus cell (nuclei count), endothelial cells (CD31^+^ cells), and macrophages (F4/80^+^ cells) in the thrombi were counted and added into five high-power fields (×400 magnification) radially into each of the three sections with 150 *μ*m interval.

### 2.8. Immunofluorescence Staining

The procedure used for immunofluorescent staining in this study was similar to that previously described [10]. Briefly, the cell smears and frozen sections of human saphenous veins and mice venous thrombi were fixed in 4% paraformaldehyde. The frozen sections of mice venous thrombi were labeled with rat antibodies against mouse Sca1, CD34, CD117, Flk1, and CD31 (Abcam, Cambridge, United Kingdom). The cell smears and frozen sections of HGSV were labeled with mouse antibodies against human CD34 (Abcam, USA) and rabbit antibody against human CD117 (Abcam, USA). The differentiated cell smears and frozen sections of venous thrombi from nude mice were labeled with rabbit antibodies against human CD31and VEGFR2 (Abcam, USA). Incubation was performed using primary antibodies for overnight at 4°C and further washed with PBS. Dylight 649 (goat anti-mouse and goat anti-rabbit) and Alexa Fluor 488 (goat anti-mouse and goat anti-rabbit) were used as secondary antibodies (Abbkine, USA), and nuclei were stained with 4,6-diamidino-2-phenylindole (DAPI). Images under fluorescence microscope (Olympus BX51, St. Louis, MO, USA) or the confocal microscopy (Olympus FV 500, Japan) were taken. Semiquantitative evaluation was performed at ×400 magnification. The number of positively stained progenitor cells in the mice venous thrombi was counted and expressed as the percentage of total nuclei.

### 2.9. Endothelial Cell Differentiation in CD34^+^CD117^+^ HGSV-AdPC *In Vitro*

The isolated CD34^+^CD117^+^ HGSV-AdPCs were seeded in Collagen IV-coated plates and cultured in endothelial growth medium (Lonza) with 50 ng/ml vascular endothelial growth factor (VEGF, Sigma) for 7 days to induce endothelial differentiation. Meanwhile, CD34^+^CD117^+^ HGSV-AdPCs were cultured in the basal medium (DMEM/F12, Lonza), and cells without growth factors served as controls. After differentiation, the cells were fixed with 4% paraformaldehyde, or extracted for gene or protein analysis by endothelial markers (CD31 and VEGFR2) using either real-time PCR, Western blotting or immunoflourescence staining. The endothelial cell differentiation was also analyzed by tube formation assay after 7 days of stimulation [12, 13].

### 2.10. Real-Time PCR

The real-time PCR procedure was similar to that described previously [10]. The total RNA was obtained from the induction group and control group by Trizol reagent (Takara China). cDNA was synthesized from the RNA using the Roche kit. The RNA level of the endothelial cell markers CD31, VEGFR2, and vWF were examined by real-time PCR. The gene-specific primers (Sheng gong, China) designed for CD31 were as follows: (5′-CCAAGATAGCCTCAAAGTCGG-3′ and 5′-CTGGGAGAGCATTTCACATACG-3′), VEGFR2 (5′-CAGACGGACAGTGGTATGGTTCTTG-3′ and 5′-GTGTCTGTGTCATCGGAGTGATATCCG-3′), vWF (5′-AAGTGTCTGGCTGAGGGAGGTAA-3′ and 5′-GTCAATGGAGTACATGGCTTTGC-3′), *β*-actin (5′-CAGGGCGTGATGGTGGGCA-3′ and 5′-CAAACATCATCTGGGTCATCTTCTC-3′). The PCR cycling conditions were as follows: 30 minutes at 42°C, 5 minutes at 95°C, 40 cycles of 30 s at 95°C for initial denaturation, and 45 seconds at 60°C for annealing/extension.

### 2.11. Western Blotting

The Western blotting procedure from cultured cells was performed with slightly modified procedure as previously described [10]. Protein extraction from induction group and control group was conducted according to the protocol of protein extraction reagent (Beyotime, China). The protein content was quantified by BCA protein assay kit (Beyotime, China). The extracted proteins (20 *μ*g) were separated on 6% sodium dodecyl sulfate-polyacrylamide gels by SDS-PAGE and then transferred onto the polyvinylidene difluoride membranes. The blots were then incubated with CD31 (1 : 500), VEGFR2 (1 : 500), and *β*-actin (1 : 1000) antibodies for overnight and then incubated with goat anti-rabbit IgG (1 : 6000) secondary antibody. To confirm equal loading of the extracted protein, the membranes were stained with antibodies against *β*-actin (Santa Cruz Biotechnology, Inc.).

### 2.12. Tube Formation Assay

*In vitro* endothelial tube formation assays were performed using a total of 10^4^ CD34^+^CD117^+^ HGSV-AdPC that were cultured in the endothelial growth medium with 50 ng/ml VEGF for 7 days. 96-well plates were coated with 50 *μ*l matrigel matrix for 45 min at 37°C. The induced CD34^+^CD117^+^ HGSV-AdPC were resuspended in endothelial growth medium containing 50 ng/ml VEGF and seeded into the matrigel matrix-coated wells. The CD34^+^CD117^+^ HGSV-AdPC cultured only in the endothelial growth medium were used as controls. The tube formation was observed under an inverse microscope after 6 hours.

### 2.13. Statistical Analysis

Statistical analyses were conducted using SPSS 12.0 through independent *t*-test. Data were expressed as means and standard deviation (*x* ± *s*) of at least three experiments. Statistically significant differences were depicted by asterisks, ^∗^*P* < 0.05, ^∗∗^*P* < 0.01.

## 3. Results

### 3.1. HGSV-AdPC

**Figure 1:**
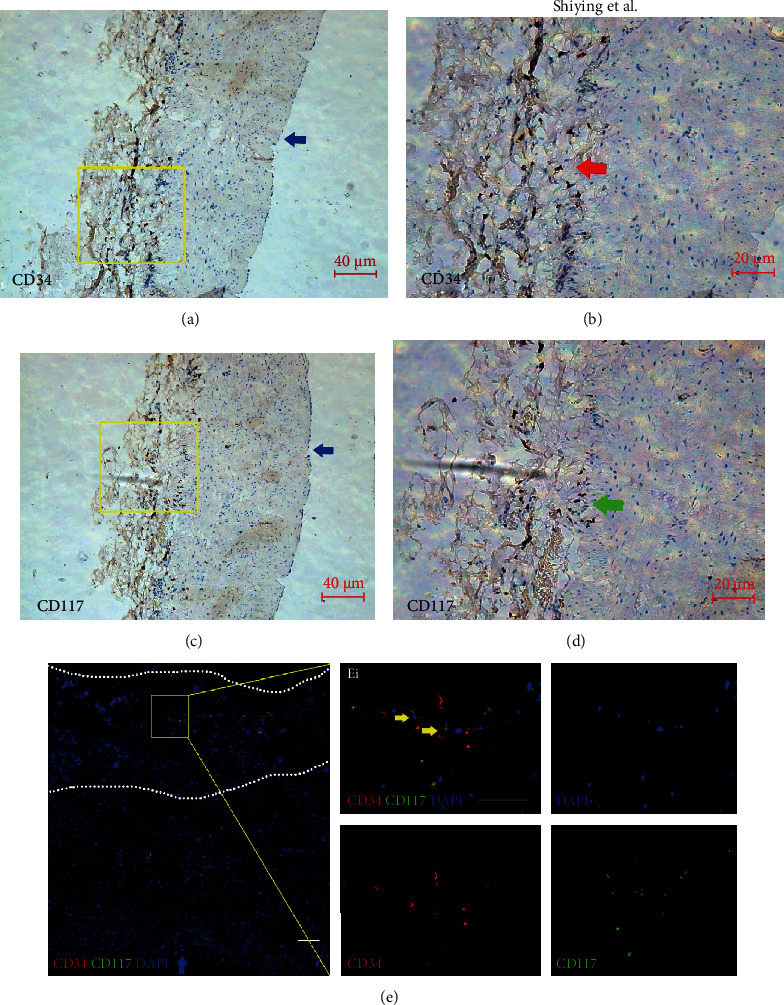
Adventitial progenitor cells in HGSV. The red arrow indicates CD34^+^cells. The green arrow indicates CD117^+^ cells. The yellow arrow indicates double staining of CD34^+^andCD117^+^ cells. The blue arrow indicates the intima of the vein. The white dotted line indicates the border of adventitia. (a, b) CD34^+^ cells in the adventitia of HGSV. (c, d) CD117^+^ cells in the adventitia of HGSV. (e) The immunofluorescent double staining of CD34^+^ and CD117^+^ cells in the adventitia of human saphenous vein. Scale bars: 40 *μ*m (a, c); 20 *μ*m (b, d); 100 *μ*m (e); 50 *μ*m (Ei). Magnification: 100x (a, c, and e), 200x (b, d), 400x (Ei).

Immunohistochemical staining showed that the CD34^+^ and CD117^+^ cells were mainly located within the adventitia of human saphenous vein, in which a few appeared in the media and seldom in the intima (Figures 1(a)–1(d)). Immunofluorescent staining of progenitor cells revealed that there were consistent, albeit small population of CD34^+^ CD117^+^ cells within the adventitia of HGSV (Figure 1(f)).

### 3.2. Progenitor Cells in Thrombi of Mice Model

**Figure 2:**
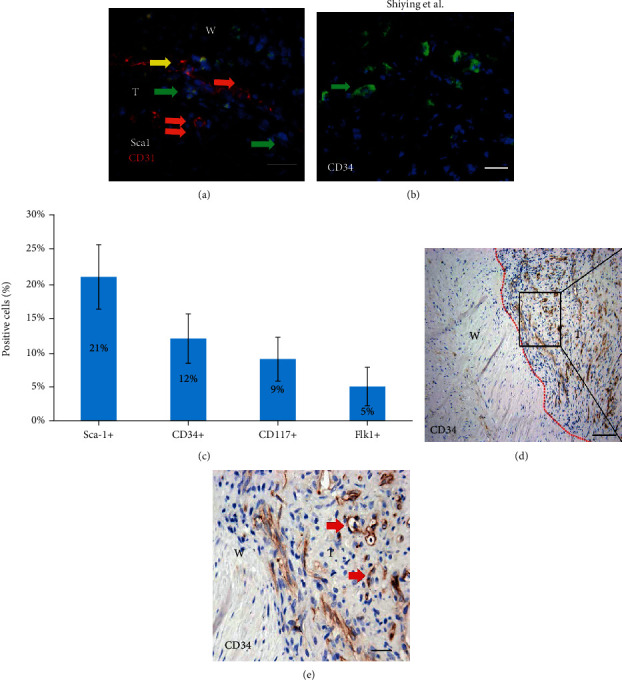
Progenitor cells in the thrombi. (a) The result of double staining with Sca1 antibody and CD31 in the 7-day thrombi of mice. The green arrow indicates Sca1^+^ progenitor cells in the venous thrombi. The red arrow indicates CD31^+^ endothelial cells in the intima of venous wall. The double red arrows indicate CD31^+^ endothelial cells of neovascularization within the marginal area of thrombi. The yellow arrow indicates crossing of Sca1^+^ progenitor cells into the endothelial cells within the intima of venous wall. (b) The labeled cells with CD34 antibody in mice. (c) Total cells and positive cells were counted under the microscope. The percentage of progenitor cells in the marginal area of thrombi from 5 mice. (d, e) The result of staining with antibody CD34 in a 25-day thrombus of HGSV. The red dotted line indicates the intima of the vein. The red arrow indicates CD34^+^ cell in thrombus. T: area of thrombi; W: area of vein wall. Scale bars: 25 *μ*m (a, b); 100 *μ*m (d); 50 *μ*m (e).

To identify the progenitor cells in the venous thrombi of mice, a thrombus mice model was established using the infrarenal inferior vena cava. Seven-day-old thrombi were harvested, and immunofluorescent staining was performed. The organization of the thrombus was mainly located at the marginal area, with no nucleolate cells in the central area of the thrombi. These are in accordance with that described previously [5]. A large number of Sca1^+^ cells were observed in the vein wall and the marginal area of thrombi. Neovascularization was also obvious within the marginal area of the thrombi. Some Sca1^+^ cells are located within the intima and were crossing the endothelial cells into the thrombi (Figure 2(a)). The Sca1^+^ cells within the vein wall have entered into the thrombi via the vasa vasorum (Suppl Figure IIA). This suggested that the progenitor cells within the adventitia of vein wall could cross the intima or via vasa vasorum into the thrombi, and neovascularization within the thrombi might be related to the accumulation of progenitor cells derived from the adventitia of vein wall. Other progenitor cell markers CD34^+^, CD117^+^, and Flk1^+^ were also observed in the vein wall and the marginal area of thrombi (Figure 2(b), Suppl Figure IA and IB). The number of progenitor cells within the marginal area of thrombi was Sca1^+^ ≈21%, CD34^+^ ≈12%, CD117^+^ ≈9%, and Flk1^+^ ≈5% (Figure 2(c)). Although the organization of 14-day-old thrombi was involved in the whole area of thrombi, the Sca1^+^ progenitor cells were mainly observed within the marginal area of the thrombi, which was similar to the location of 7-day thrombi (Suppl Figure IIB-IIE).

### 3.3. Progenitor Cells in Thrombi of HGSV

To identify the progenitor cells in the thrombi of human veins, patients with great saphenous vein varicose and superficial thrombophlebitis underwent surgery for harvesting the HGSV thrombi and the vein wall. The thrombi from 5 patients with a history of thrombus symptoms varying from 23-26 days were examined. The results of immunohistochemical staining revealed that the whole thrombi have completed organization in all five patients, and many CD34^+^ and CD117^+^ cells have appeared in the whole area of thrombi (Figures 2(d) and 2(e), Suppl Figure IC and ID). However, it is unclear whether the CD34^+^ and CD117^+^cells in the thrombi were derived from the adventitia of HGSV.

### 3.4. The Cells Outside-In Migration into Decellularized Vessel Wall

**Figure 3:**
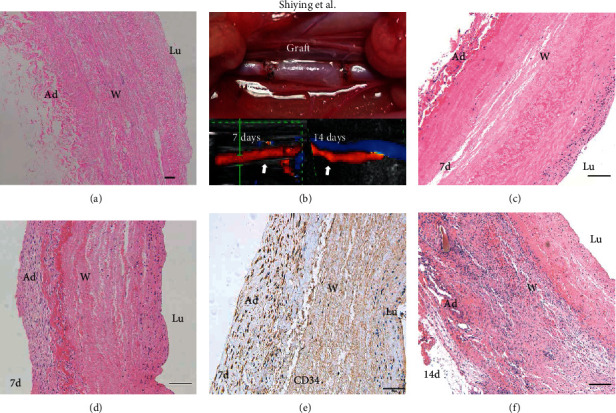
The cells outside-in migration into decellularized HGSV. (a) No cell nuclei was visible with HE staining of decellularized vessels. (b) The infrarenal abdominal aorta of rabbit was replaced by decellularized HGSV. The white arrow indicates the patented transplants by ultrasonic examination at 7-day and 14-day replacement. (c) At 7-day replacement, there were a small number of cells in the adventitia of scaffold far from anastomoses; meanwhile, there were several cells emerged from the intima and seldom in tunica media. (d) At 7-day replacement, there were a large number of cells in the adventitia of scaffold close to anastomoses; meanwhile, a large number of cells have emerged in the intima and a few in the tunica media. (e) A large amount of CD34^+^cells in the adventitia of scaffold and a small number of CD34^+^ cells in the intima. (f) Cells entered into the three layers of the scaffold at 14-day replacement. Ad: adventitia of scaffold; W: wall of scaffold; Lu: lumen of scaffold. Scale bars: 25 *μ*m (a); 50 *μ*m (c, d, and f); 100 *μ*m (e). Magnification: 100x (b, c, d, and f); 200x (e).

The HGSV was decellularized by treatment with 0.75% sodium dodecyl sulfate for 8 hours and washed with PBS. Then, translucent acellular vessel scaffolds were generated, and HE staining of these vessels showed no cell nuclei (Figure 3(a)). The decellularized vessels were used to replace the rabbit infrarenal abdominal aorta. The graft kept patency at 7- and 14-day follow-up was evaluated with ultrasonic examination, and no thrombi were found in the lumen of the graft (Figure 3(b)). This was controversial with that of the outside-in theory with regard to migration of cells in the resolution of venous thrombi. It was unclear as to whether the cells entered into the thrombi from vein wall or circulation. Hence, in this study, we aimed to evaluate the migration of cells into the acellular scaffold and the expression of CD34 marker. Interestingly, at 7-day replacement, there were small number of cells in the adventitia at the area of scaffold far from anastomoses (Figure 3(c)); meanwhile, a large number of cells have emerged in the adventitia at area of scaffold next to anastomosis (Figure 3(d)). Almost similar number of cells appeared in the intima and a few cells in the tunica media of scaffold at both areas. Furthermore, most of the cells in the adventitia of scaffold far to anastomoses were CD34 positive, and a small number of cells in the intima were CD34 positive (Figure 3(e)). However, the cells completely migrated into the three layers of the scaffold both at the sites of far from anastomoses and central portion at 14-day replacement (Figure 3(f)). These findings suggested that the cells migrated into the decellularized vessels probably mainly by originating from the adventitial progenitor cells of the abdominal aorta of the rabbit, and the outside-in migration was the main mechanism of vessel remodeling. Especially when the thrombi occlude the venous lumen, then the cells enter into the venous thrombi most probably through outside-in migration.

### 3.5. Isolation of CD34^+^CD117^+^ HGSV-AdPC *In Vitro*

**Figure 4:**
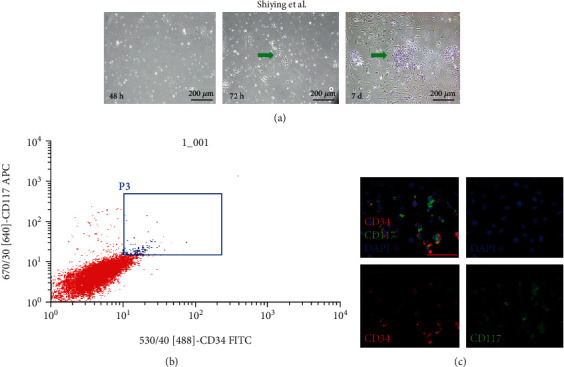
Isolation and cultivation of CD34^+^CD117^+^HGSV-AdPC *in vitro*. (a) Cells of HGSV wall obtained by collagenase II digestion at 48 hours, 72 hours, and 7 days culture. The green arrow indicates cells growing in colony. (b) FACS isolation of CD34^+^CD117^+^HGSV-AdPC; the double positive cells were about (1.2 ± 0.07)% in vein wall cells. (c) Immunofluorescent staining with antibodies CD34 (red), CD117 (green), and DAPI (blue) of the FACS-purified HGSV-AdPC after 7 days culture, and the double-positive cells accounted for 91.2%. Scale bars: 200 *μ*m (a), 50 *μ*m (c). Magnification: 100x (a), 400x (c).

To investigate the functions of HGSV-AdPC, the adventitial CD34^+^CD117^+^ HGSV-AdPC were isolated from the primary cultures. The HGSV was minced, and the vascular wall cells were obtained by collagenase digestion. The harvested vascular wall cells began to adhere to the flask in 48 hours culture, and grew in a colony in 72 hours culture. After 7 days culturing, the number of cells was abundantly increased and the colonies were increased in size as well as number. Some vascular wall cells appeared as spindle, triangular, and irregular shapes (Figure 4(a)). At third generation, approximately 10^7^ vascular wall cells were gathered, and then, CD34^+^CD117^+^ HGSV-AdPC (1.2 ± 0.07)% were isolated by FACS (Figure 4(b)). To confirm the purity obtained by FACS, the target cells were stained by immunofluorescence after 7-day culturing, and double positive cells accounted for 91.2% (Figure 4(c)).

### 3.6. Transplantation of CD34^+^CD117^+^ HGSV-AdPC in the Adventitia-Enhanced Resolution of Venous Thrombi

**Figure 5:**
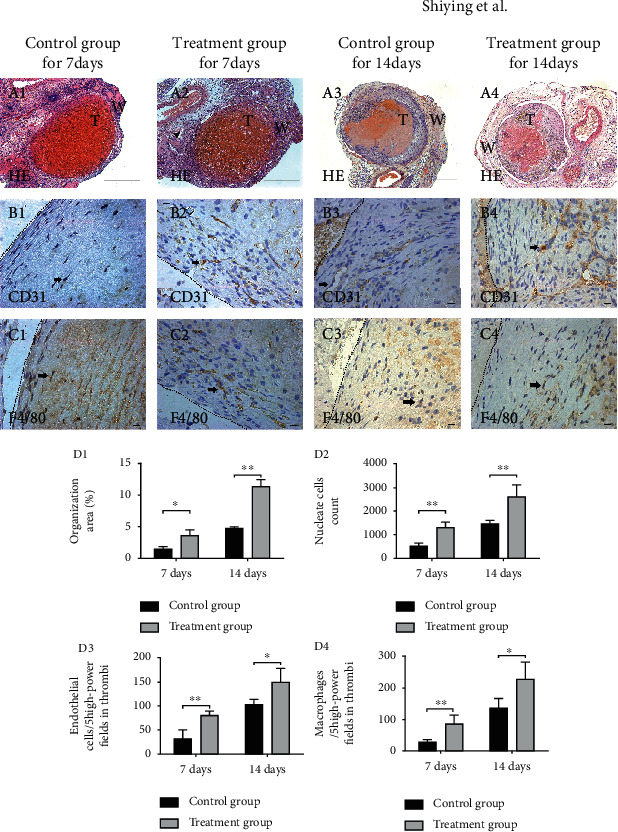
The organization and neovascularization of thrombi in control and treatment groups at postoperative days 7 and 14 (*n* = 6). T indicates thrombus, and the black arrow indicates positive cells. A1–A4, The HE staining, and A indicates artery. B1–B4, Immunohistochemical staining with CD31 antibody. C1–C4, Immunohistochemical staining with F4/80 antibody. As venous thrombi organization progressed, the monocytes and the endothelial cells entered into the thrombus, wherein the neovascularization appeared. D1–D4, The organization area, thrombus cell counts, the number of endothelial cells, and macrophages in control and treatment groups, respectively. ^∗^Significant differences between control and treatment group, *P* < 0.05. ^∗∗^Significant differences between control and treatment group, *P* < 0.01. T: area of thrombi; W: area of vein wall. Scale bars: 100 *μ*m (A1–A4); 15 *μ*m (B1–C4). Magnification: 40x (A1–A4), 400x (B1–C4).

To clarify whether adventitial CD34^+^CD117^+^HGSV-AdPC cells play a role in the resolution of venous thrombosis, the HGSV-AdPC harvested from the culture *in vitro* were transplanted into the adventitia of nude mice infrarenal vena cava to induce thrombosis. On posttransplantation days 7 and 14, the infrarenal vena cava was harvested, and histochemical staining and cell markers (CD31, F4/80) were immunohistochemically stained to detect the organization, neovascularization, nucleate cell, and macrophage infiltration within the thrombi. The organization rate of the treatment group (43.3% ± 8.7) was significantly different from that of the control (31.1 ± 5.6%, *P* < 0.05) on posttransplantation day 7 and was 81.2 ± 11.5% versus 60.5 ± 10.6% (*P* < 0.01) on posttransplantation day 14, respectively (Figures 5A1–4 and 5D1). The number of nucleate cells in thrombi on day 7 in the control and treatment groups was 505.3 ± 136.7 vs. 1306.7 ± 223.7 (*P* = 0.001) and 1446.7 ± 162.3 vs. 2595.5 ± 522.2 (*P* = 0.002) on day 14 (Figures 5A1–4 and 5D2). The endothelial cells of the thrombi in the treatment group has dramatically accelerated when compared with the control group on day 7 (31.4 ± 18.2 vs. 79.6 ± 9.4, *P* = 0.006) and on day 14 (102.8 ± 11.0 vs. 148.6 ± 28.4, *P* = 0.04) (Figures 5B1–4 and 5D3). Macrophage cells (F4/80) were significantly increased in the treatment group on day 7 (28.4 ± 8.7 vs. 86.2 ± 28.8, *P* = 0.003) and on day 14 (135.2 ± 31.6 vs. 228.6 ± 54.5, *P* = 0.025) (Figures 5C1–4 and 5D4). These results showed that the increase in adventitial CD34^+^CD117^+^HGSV-AdPC could promote thrombus organization and accelerate thrombi resolution, neovascularization, and recruitment of macrophage within the venous thrombi.

### 3.7. Transplanted CD34^+^CD117^+^HGSV-AdPC at Adventitia Crossed Vein Wall, Entered Thrombi, and Differentiated into Endothelial Cells of Neovascularization

**Figure 6:**
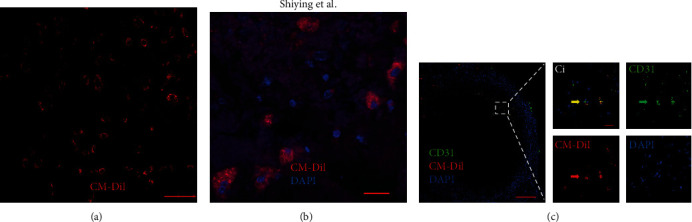
The migration and differentiation of labeled CD34^+^CD117^+^HGSV-AdPC in 7-days thrombus of mice. (a) CD34^+^CD117^+^HGSV-AdPC labeled with fluorescent molecular probe (CM-Dil) before transplantation. (b) The CM-Dil-labeled CD34^+^CD117^+^HGSV-AdPC entered into thrombus. (c) The labeled CD34^+^CD117^+^HGSV-AdPC differentiated into CD31^+^ endothelial cells. Scale bars: 20 *μ*m (a, b and Ci); 200 *μ*m (c). Magnification: 200x (a); 400x (b); 100x (c).

To clarify the mechanism of thrombi resolution after transplantation of adventitial progenitor cells, the CD34^+^CD117^+^HGSV-AdPC that were cultured *in vitro* detected by FACS had 82.5 ± 2.9% purity (Suppl Figure IIIA) and were labeled with CM-Dil *in vitro* (Figure 6(a)). The labeled cells were transplanted into the adventitia of infrarenal vena cava of nude mice, and the thrombosis was induced. The infrarenal vena cava was harvested on day 7 or 14 of the labeled cells transplantation. On day 7, many CM-Dil-labeled cells were observed within the vein wall, and some CM-Dil labeled cells have crossed the vein wall and gathered in the marginal area of venous thrombi (Figure 6(b) and Suppl Figure IIIB). These suggested that the labeled cells transplanted into the adventitia of infrarenal vena cava were migrated by outside-in process via vein wall and entered into the thrombi. To further explore the role of CD34^+^CD117^+^HGSV-AdPC in thrombi, the labeled cells in thrombi were tested by immunofluorescent staining with antibodies of endothelial cell markers (CD31 and VEGFR2) after labeled cells were transplanted on day 7. Most of the CM-Dil labeled cells within the thrombi expressed CD31 (Figure 6(c)) and VEGFR2 (Suppl Figure IIIC), suggesting that the CM-Dil-labeled cells in the thrombi differentiated into endothelial cells.

### 3.8. CD34^+^CD117^+^ HGSV-AdPC Differentiated into Endothelial Cells *In Vitro*

**Figure 7:**
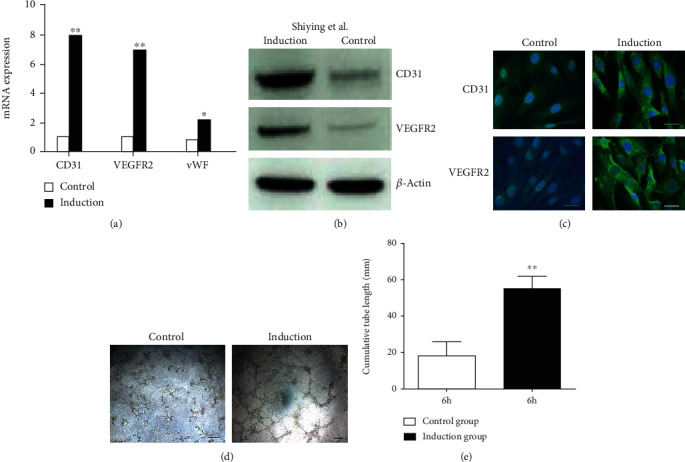
CD34^+^CD117^+^HGSV-AdPCs differentiated into endothelial cells *in vitro*. CD34^+^CD117^+^HGSV-AdPCs were isolated with FACS and cultured in the presence of VEGF for 7 days. (a) The mRNA expression of CD31, VEGFR2, and vWF. (b) The result of Western blotting of CD31 and VEGFR2 proteins in the induction and control groups. (c) The differentiation result of immunofluorescent staining with antibodies CD31 and VEGFR2 in control and induction groups. (d) The tube formation observed under inverse microscope at 6 hours culture in control and induction groups. (e) The difference of tube formation at 6 hours culture between control and induction groups. Scale bars: 10 *μ*m (c); 200 *μ*m (d). Magnification: 200x (a, d).

After the adventitial progenitor cells cross the vein wall, they enter into thrombi and differentiate into endothelial cells *in vivo*, and so we attempted to explore the differentiation of CD34^+^CD117^+^ into endothelial cells *in vitro*. FACS was used to isolate CD34^+^CD117^+^ HGSV-AdPC from primary cultures, and the cells were subjected to endothelial cell differentiation in the culture medium in the presence of VEGF. Using real-time PCR, we found that VEGF showed significant gene expression of endothelial cell markers (CD31, VEGFR2, and vWF) (Figure 7(a)). The induction of endothelial cell differentiation at protein level was confirmed by Western blotting (Figure 7(b)) and immunofluorescent staining (Figure 7(c)), and the protein expression of endothelial cell markers (CD31 and VEGFR2) was significantly increased when compared to the control. The significant difference of tube formation was also observed under inverse microscope at 6 hours culture between the control and induction groups (Figures 7(d) and 7(e)). These results demonstrated that tCD34^+^CD117^+^ HGSV-AdPC have the potential to differentiate into endothelial cells in endothelial growth media in the presence of VEGF.

## 4. Discussion

Over the last decade, the vascular wall progenitor cells have been identified in the vessel wall of both rodents as well as humans [8–10, 14–18]. The adventitia of the vessel wall has been established as a niche for progenitor cells with the potential to differentiate into several types of mature cells to regulate and initiate vascular remodeling and disease development, especially atherosclerosis and intimal hyperplasia [10, 18, 19]. However, there were currently no reports on the role of adventitial progenitor cells in the resolution of venous thrombosis. This is the first study to show that the HGSV-AdPC has migrated in an outside-in process via the vein wall into the thrombi of the lumen, and promoted the resolution of venous thrombi. HGSV-AdPC had potential to differentiate into endothelial cells both *in vivo* and *ex vivo*. The adventitial progenitor cells play an important role in the natural resolution of venous thrombi. The underlying mechanism of this process involves neovascularization induced by adventitia-derived progenitors via differentiation of endothelial cells.

Non-bone marrow progenitor cells are clinically unexploited due to their scarcity. In accordance with the previous findings [9, 20–22], many CD34^+^ and CD117^+^ progenitor cells were observed in the adventitia of HGSV, and some of the progenitors were CD34 and CD117 double positive. Stripping of HGSV is a common surgical method in clinical practice in patients with HGSV varicose. Therefore, the disposed saphenous vein from patients with great saphenous vein varicose could be collected as the source of progenitor cells. The progenitor cells were involved in the resolution of venous thrombosis. Mobilization with subcutaneous injection of granulocyte colony-stimulating factor enhanced organization and recanalization of thrombi, and the neovascularization and macrophage number of the thrombi were increased [5]. However, the derivation of the progenitors in thrombi remained unclear. Bone marrow-derived cells were recruited into the thrombi during resolution, but the cells did not line the new recanalizing channels within the thrombi [7]. Similar instance also occurred in other vascular diseases. Hu et al. have revealed that the progenitor cells in the atherosclerosis and the neointimal lesion of vessel allografts were not derived from the bone marrow of progenitor cells but local host cells [23]. Iwata et al. have shown that the bone marrow cells only contribute to inflammation of the vessel but do not differentiate into smooth muscle cells (SMC) in wire injury-induced neointima [24]. How the adventitial progenitor cells move into the intima is still equivocal.

The adventitial progenitor cells might first enter into the blood circulation via vasa vasorum and then move into the intima from blood circulation. However, there were no vasa vasorum for decellularized vessel, and found that the progenitors appeared both in vein wall and thrombi, and some progenitors within the intima crossed the endothelial cells into the thrombi. After the decellularized HGSV replaced the rabbit infrarenal abdominal aorta, there were more adventitial progenitor cells close to anastomosis than that far from anastomoses on postreplacement day 7, but there were similar number of cells in the intima of areas far from and close to anastomoses, and a few cells in the tunica media. The cells were migrated into all three lays of decellularized vein both at the areas close to or far from anastomoses on postreplacement day 14. These results indicated that adventitial progenitor cells in decellularized vein might migrate from the adventitia of host abdominal aorta and accumulate in a time-dependent manner in the tunica media, while the cells in the intima were derived from the circulation. However, when the venous thrombosis occurs, then the thrombi occlude the lumen, and no blood flow was observed within the lumen. After that, the adventitial progenitor cells migrate into the thrombi via the tunica media and intima. This outside-in migration process was similar to the previously published data, which showed that the mobilization and recruitment of adventitial progenitor cells were responsible for intimal repair during vascular remodeling such as intimal injury [14, 25–27]. The outside-in migration of adventitial progenitor cells was also responsible for neointimal formation and atherosclerosis of decellularized vessel. The adventitia of vein grafts containing a considerable number of progenitor cells was shown to migrate from the external side into the decellularized vessel in response to SDF-1 [10]. The study conducted by Wong et al. have demonstrated that the progenitor cells migrate from the adventitia into the intima and then seeded into the adventitial Sca1^+^ cells on the outside of a decellularized vessel migrated to the inner side of the vessel in response to sirolimus [19]. Tsai et al. have explored that the adventitial progenitor cells contribute to the neointimal formation in decellularized vessel grafts and found Sca1^+^, c-Kit^+^, and CD34^+^ cells in the neointima [28]. Adventitial progenitor cells not only migrate from the adventitia into the intima of decellularized vessel both *in vitro* and *in vivo* but also migrate from the adventitia into the intima of artery wall *in vivo* [15, 25]. The CM-Dil-labeled HGSV-AdPC were seeded outside the infrarenal vena cava, in which the thrombi were induced, and the labeled progenitor cells migrated from the adventitia into the thrombi and differentiated into the endothelial cells of neovascularization in thrombi. So, the adventitial progenitor cells moved into the intima or venous thrombi not via circulation, but via crossing the vein wall. Yu et al. have found that the adventitial progenitor cells migrate from the adventitia to the neointima by inducing the chemokines (CCL2 and CXCL1) [27]. In our previous study, the stem/progenitor cells mobilize with granulocyte colony-stimulating factor to enhance the resolution and recanalization of venous thrombi by increasing CCR2 expression [5]. The resolution of venous thrombi involves CXCR2-mediated neovascularization, fibrinolysis, and collagen turnover, and this process is probably monocyte-dependent [29]. Targeted deletion of CCR2 in the mice model of venous thrombosis showed association with impairment of thrombi resolution [30]. So, the migration of adventitial progenitor cells from the adventitia into the thrombi might be associated with CCL2/CCR2.

Neovascularization in the thrombi is shown to be highly related to the resolution of venous thrombosis [5]. Our research showed that the CD34^+^CD117^+^ HGSV-AdPC could differentiate to endothelial cells *in vitro*. The progenitor cells harvested from the culture *in vitro* were seeded outside the nude mice inferior vena cava, in which the thrombi were shown to be induced, and the organization, neovascularization, nucleate cells, and macrophage infiltration within the thrombi were highly promoted. This indicated that the increase in the number of adventitial progenitor cells could enhance the resolution of venous thrombi. Previous studies have also shown that the HGSV-AdPC differentiated into endothelial cells and boosted neovascularization. Campagnolo et al. have investigated the HGSV-AdPC and showed the clonogenic and multilineage differentiation capacities, integration into networks of endothelial cells, and neovascularization through paracrine mechanisms [9]. Iacobazzi et al. have evaluated the effects of oxidative stress on the viability and functionality of HGSV-AdPC and found that stress does not impair the capacity of neovascularization of progenitor cells, and an injection to the ischemic tissue helps perfusion recovery and neovascularization [20]. Gubernator et al. have transplanted HGSV-AdPC into the ischemic limbs and showed improvement of the revascularization of ischemic limbs and highlighted the angiogenic network [21]. Although endothelia progenitors have been identified in the GSV intima and demonstrated to have vasculogenic potential [31], our results showed that CD34^+^ and/or CD117^+^ stem cells were almost all located within the GSV adventitia. Therefore, even if there were a few CD34^+^ and CD117^+^ double-positive stem cells in the intima, the effects on our study and conclusion were limited. Of course, a potential role of luminal resident endothelial progenitors in the models cannot be completely overruled. To the best of our knowledge, our study is the first to provide direct evidence that the adventitial progenitor cells migrate into the venous thrombi and differentiate into endothelial cells of neovascularization. The differentiation of adventitial progenitor cells is regulated by local environment [32].

We found that the organization was involved in the whole area on day 14 venous thrombi in normal mice, but the organization rate was 60.5 ± 10.6% in 14-day venous thrombi of nude mice and 81.2 ± 11.5% in nude mice with CD34^+^CD117^+^ HGSV-AdPC transplantation. This might be related to the lower inflammation in the thrombi of nude mice. Myocardial damage is shown to be less prominent and a mild mononuclear cell infiltration in nude mice when compared with normal mice [33, 34]. Our results showed that C34^+^ and CD117^+^ progenitor cells have emerged into the whole organization area in humans on day 23-26 thrombi, but the Sca1 progenitor cells have appeared only in the marginal organization area of mice on day 14 thrombi. Although the organization was found to be involved in the whole area in the 14-day thrombi in normal mice, the Sca1^+^ progenitor cells appeared mainly within the marginal area of the thrombi, which was similar to the area of 7-day thrombi. These reasons might be related to the differences in thrombi history and the varieties of progenitor cells between humans and mice in the present study. There were various kinds of progenitor cells present in the adventitia, such as CD34^+^, CD117^+^, Sca1^+^, NG2^+^, CD146^+^, CD44^+^, and Sox2^+^ [35], and their fate of differentiation might be different. Depending on the environmental cues, the adventitial progenitor cells might express cell fates that resemble the tissue resident cells, including the osteoblasts, endothelial-like cells, macrophages, mural cells, and adipocytes. There was heterogeneity of adventitial Sca1^+^ cells, and the aortic adventitia contains at least two types of Sca1 progenitor cells, and one express a myogenic phenotype whereas the other expresses a macrophage phenotype.

In the present study, the adventitial progenitor cells promoted resolution of thrombi, macrophage accumulation, and neovascularization. This was similar to that of the previous studies, wherein the increase of macrophage accumulation in thrombi showed association with the resolution of thrombi [5], the injection of macrophages within the thrombi resulted in fivefold decreased thrombi size and fourfold increased neovascularization when compared with controls [36]. Neovascularization of thrombi is mostly shown at the areas with macrophage accumulation [37]. However, it is unclear as to how the macrophages were involved in neovascularization in the thrombi. Macrophages were the main source of tumor necrosis factor-*α* (TNF-*α*), which play a role in mediating neovascularization, VEGF production, and macrophage outgrowth. The proangiogenic effects of TNF-*α* was suppressed by blocking VEGF. TNF-*α*/VEGF-mediated angiogenic pathway requires macrophages [38]. Wong et al. have demonstrated that the macrophages assist in inducing differentiation of adventitial progenitor cells into endothelial cells and inhibit SMC differentiation. Macrophage-derived TNF-*α*-mediated endothelial cell differentiation occurs by binding to nuclear factor-*κ*B (NF-*κ*B). The knockout of TNF-*α* in a vein graft model decreased reendothelialization. Macrophage culture media contains angiogenic factors including VEGF-A and VEGF-C [39]. VEGF and basic fibroblast growth factor were found in the resolving thrombi [40]. Adenovirus-mediated transfection of VEGF treatment reduced the size of the thrombi and increased recanalization and macrophage recruitment into the thrombi. This process is probably due to the increase of macrophage recruitment [41]. Hypoxia and hypoxia-inducible factor 1 (HIF-1) were induced in the naturally resolving thrombi and showed correlation with increased VEGF expression. Upregulation of HIF-1 enhanced thrombi resolution and vein recanalization [42]. Our results demonstrated that VEGF promotes the expression of endothelial cell markers of adventitial progenitor cells at both gene and protein levels. Tube formation was also more obvious in the culture media containing VEGF. These results showed that the macrophages in thrombi might induce adventitial progenitor cell differentiation into the endothelial cell of neovascularization, and VEGF might be involved in the process.

In this study, the decellularized HGSV were used as scaffold to replace rabbit infrarenal abdominal aorta. There were no anticoagulants and other specific drugs used after operation. The graft kept patency at follow-up days 7 and 14 with ultrasonic examination, and no thrombi and stenosis were observed in the lumen of the graft. No blood leakage was observed at the anastomotic site and graft. The cells, including the adventitial progenitor cells, migrated into the decellularized scaffold and promoted vessel remodeling. The scaffold of decellularized HGSV demonstrated excellent vessel function. Bai et al. have found that hyaluronic acid-conjugated heparin-coated HGSV decreased neointimal thickness when used in venoplasty and arterioplasty in rat [43]. Kumar et al. have used decellularized HGSV that connects to a bioreactor and perfuse with endothelial medium and fresh blood. HE-staining showed that recellularization of decellularized HGSV in the presence of cells on the luminal surface of the vein [44]. Decellularized HGSV is a promising vessel scaffold.

In summary, the adventitia of HGSV contains a large amount of progenitor cells that could migrate from the adventitia of venous wall into the venous thrombi and play a role in the resolution of thrombi. The progenitor cells within the venous thrombi are obtained from venous adventitia and differentiate into endothelial cells of neovascularization. Increased venous adventitial progenitor cells could promote the resolution of venous thrombi. Our results illustrated the mechanism of natural resolution of venous thrombi and clarified the role of adventitial progenitor cells in thrombi resolution. These results are encouraging for new perspectives of cell therapy approaches that target adventitial progenitor cells to enhance the resolution of venous thrombi. Meanwhile, the disposed HGSV after surgery from patients with great saphenous vein varicose could be harvested as a source of progenitor cells and vessel scaffold.

## Data Availability

The figures in the manuscript can be found as underlying data supporting the results of our study.

## Supplementary Materials

Supplementary 1 Figure I: progenitor cells in thrombi. A, B The positive stem cells were labeled with CD117 and Flk1 antibodies in 7-day thrombi of mice. C, D The result of staining with antibody CD117 in a 25-day thrombus of HGSV. Scale bars: 25 *μ*m (A, B); 100 *μ*m (C); 50 *μ*m (D). Magnification: 400x (A, B, and D); 100x (C).

Supplementary 2 Figure II: progenitor cells in days 7 and 14 venous thrombi of mice. The result of immunofluorescent staining with antibody Sca1. The green arrow indicates Sca1+ cells, and the blue arrow indicates nucleus. A The result of immunofluorescent staining with antibody Sca1 on day 7. The Sca1+ cells within the vein wall entered into the thrombi via the vasa vasorum. The red arrow indicates endothelial cells of the intima in venous wall. B The result of immunofluorescent staining with antibody Sca1 on day 7. C The merged staining result of nucleus, Sca1 antibody, and CD31 antibody on day 7. The red arrow indicates the endothelial cell of intima in the venous wall. Neovascularization appeared in the peripheral area of venous thrombi. D The result of immunofluorescent staining with antibody Sca1 on day 14. E The merged staining result of nucleus and antibody Sca1 on day 14. Dotted lines indicate the border between intima and thrombi. There were seldom cells in the central area on day 7 venous thrombi, while many cells in the central area on day 14. However, Sca1+ progenitor cells mainly appear in the venous wall and peripheral area of venous thrombi on days 7 and 14. T: area of thrombi; W: area of vein wall. Scale bars: 25 *μ*m (A); 100 *μ*m (B–E). Magnification: 400x (A); 100x (B–E).

Supplementary 3 Figure III: the migration and differentiation of labeled CD34+CD117+HGSV-AdPC in the thrombus of mice. A The purity of CD34+CD117+HGSV-AdPC detected by FACS (83.5 ± 2.9)% before transplantation. B The CM-Dil-labeled CD34+CD117+HGSV-AdPC enters into the thrombus at posttransplantation day 7. C The result of immunofluorescent staining with antibody VEGFR2. The green arrows indicate VEGFR2+ cells. The red arrows indicate CM-Dil-labeled CD34+CD117+HGSV-AdPC. The yellow arrow indicates transplanted CD34+CD117+HGSV-AdPC differentiation into endothelial cells. Scale bars: 200 *μ*m (B); 100 *μ*m (C); 25 *μ*m (Merged). Magnification: 100x (B, C); 400x (Merged).
