# Supplementary figures and images for: Adventitial Progenitor Cells of Human Great Saphenous Vein Enhance the Resolution of Venous Thrombosis via Neovascularization
Source: Stem Cells Int. 2021 Feb 23;2021:8816763. doi: 10.1155/2021/8816763 (PMC7926266; doi:10.1155/2021/8816763)

Figure I.

Shiying et al.

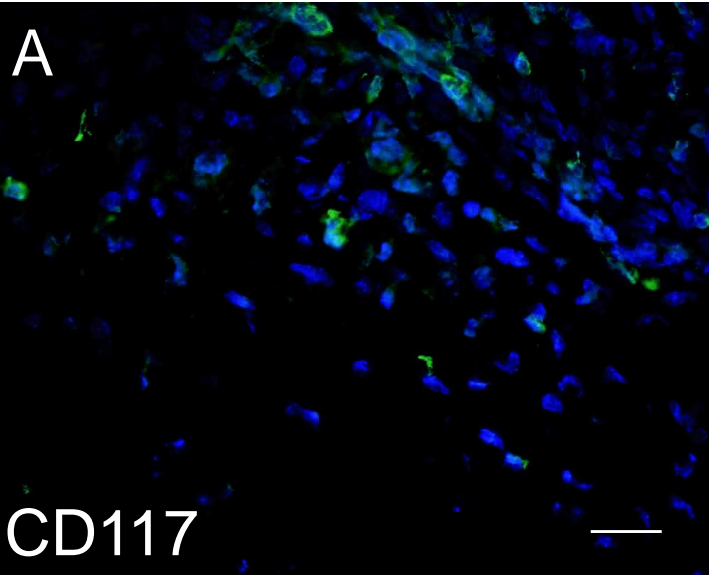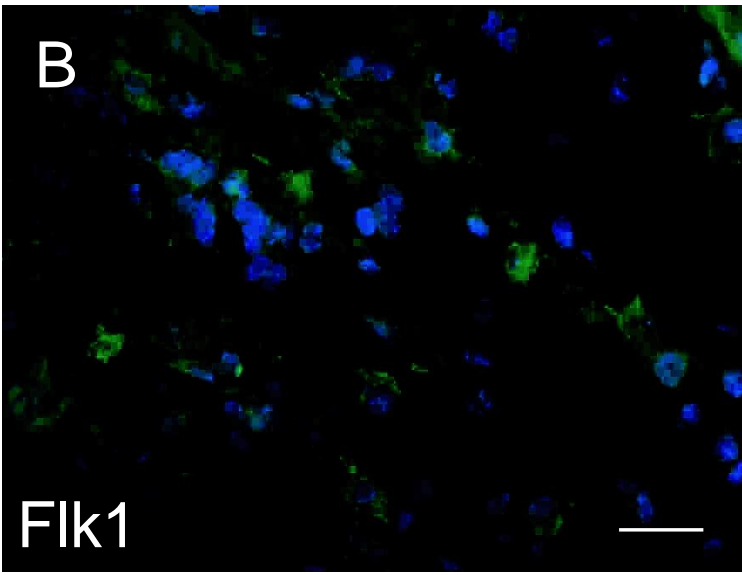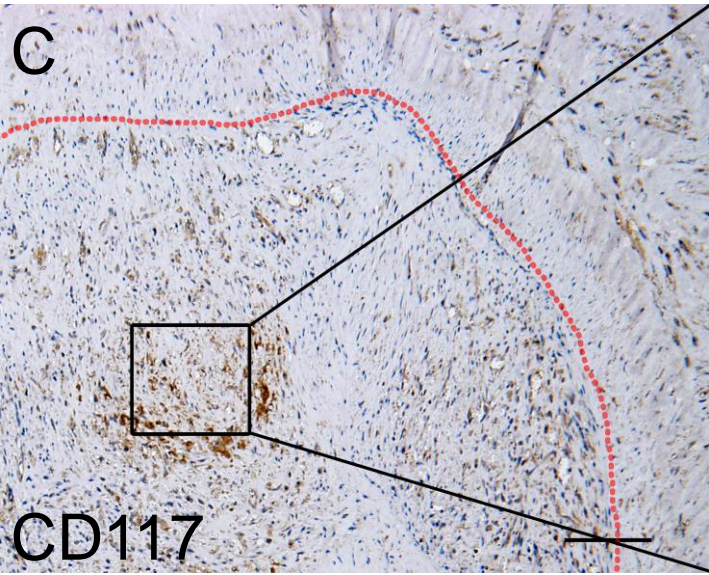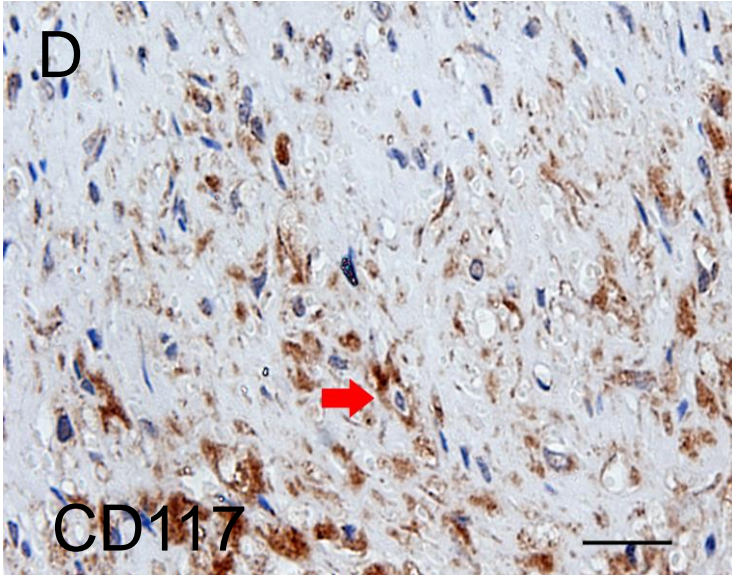

Supplement: Supplementary 1 — Figure I: progenitor cells in thrombi. A, B The positive stem cells were labeled with CD117 and Flk1 antibodies in 7-day thrombi of mice. C, D The result of staining with antibody CD117 in a 25-day thrombus of HGSV. Scale bars: 25 μm (A, B); 100 μm (C); 50 μm (D). Magnification: 400x (A, B, and D); 100x (C). [file 8816763.f1.pdf]

Figure II.

Shiying et al.

A

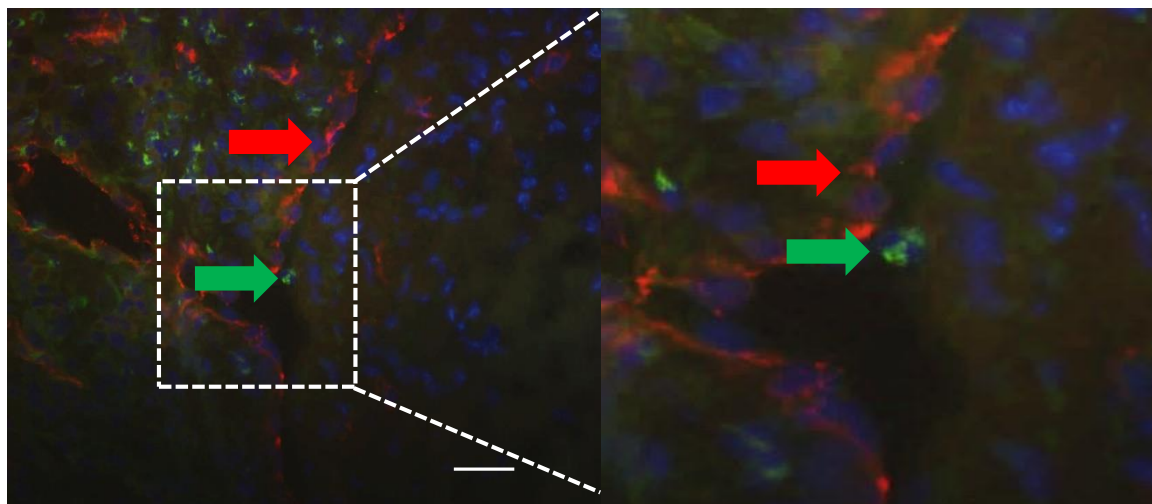

B

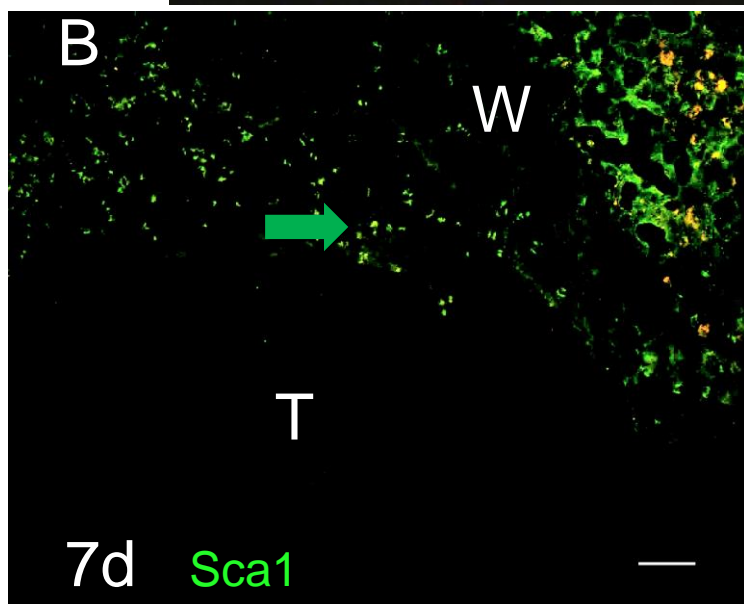

C

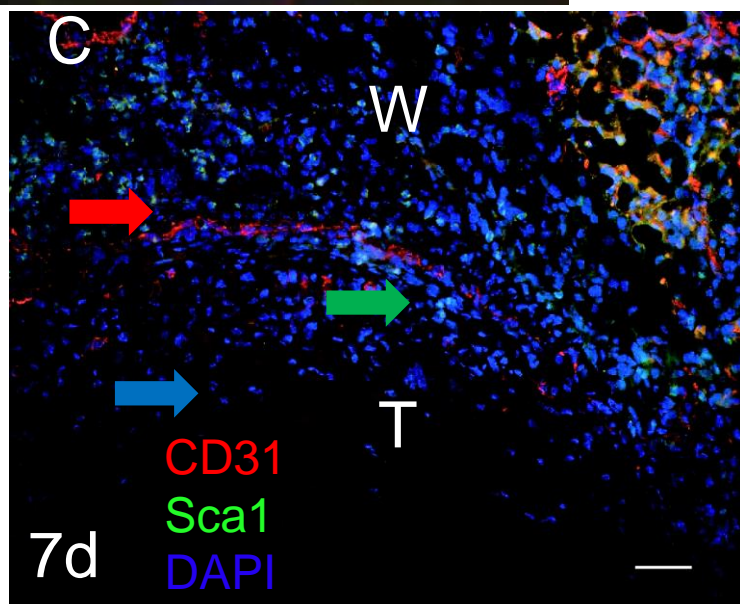

D

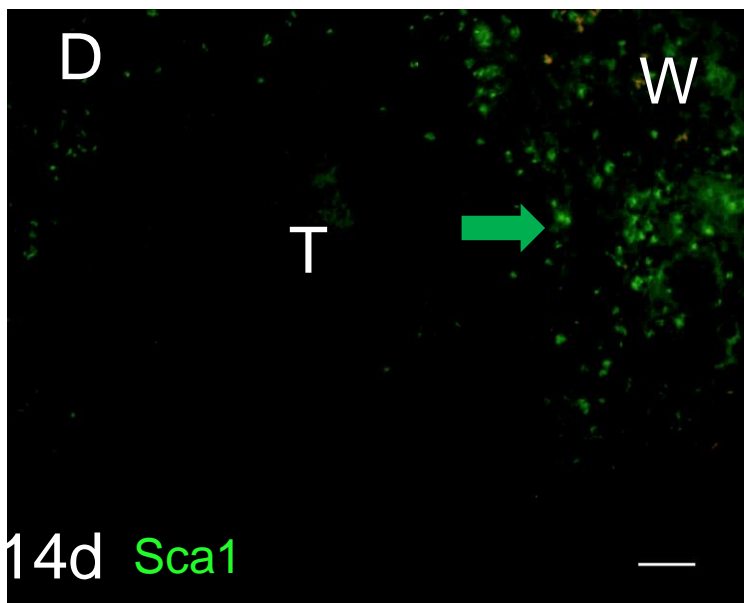

E

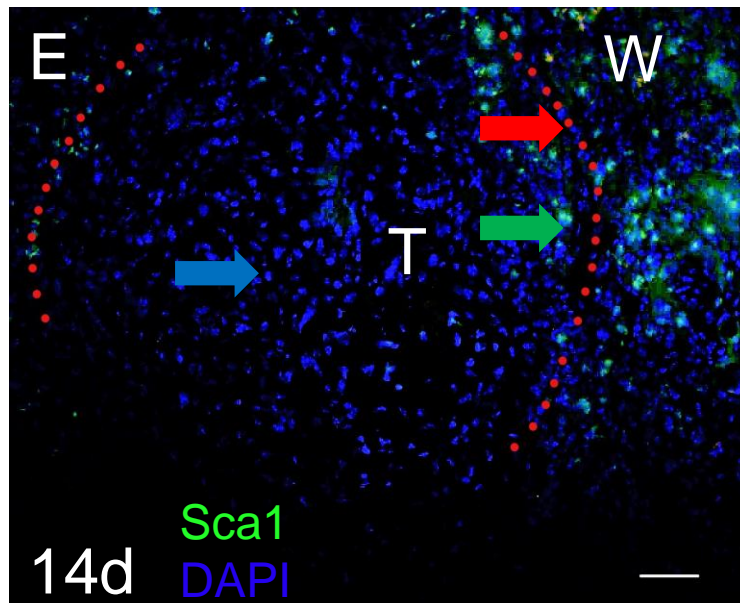

Supplement: Supplementary 2 — Figure II: progenitor cells in days 7 and 14 venous thrombi of mice. The result of immunofluorescent staining with antibody Sca1. The green arrow indicates Sca1+ cells, and the blue arrow indicates nucleus. A The result of immunofluorescent staining with antibody Sca1 on day 7. The Sca1+ cells within the vein wall entered into the thrombi via the vasa vasorum. The red arrow indicates endothelial cells of the intima in venous wall. B The result of immunofluorescent staining with antibody Sca1 on day 7. C The merged staining result of nucleus, Sca1 antibody, and CD31 antibody on day 7. The red arrow indicates the endothelial cell of intima in the venous wall. Neovascularization appeared in the peripheral area of venous thrombi. D The result of immunofluorescent staining with antibody Sca1 on day 14. E The merged staining result of nucleus and antibody Sca1 on day 14. Dotted lines indicate the border between intima and thrombi. There were seldom cells in the central area on day 7 venous thrombi, while many cells in the central area on day 14. However, Sca1+ progenitor cells mainly appear in the venous wall and peripheral area of venous thrombi on days 7 and 14. T: area of thrombi; W: area of vein wall. Scale bars: 25 μm (A); 100 μm (B–E). Magnification: 400x (A); 100x (B–E). [file 8816763.f2.pdf]

Figure III.

Shiying et al.

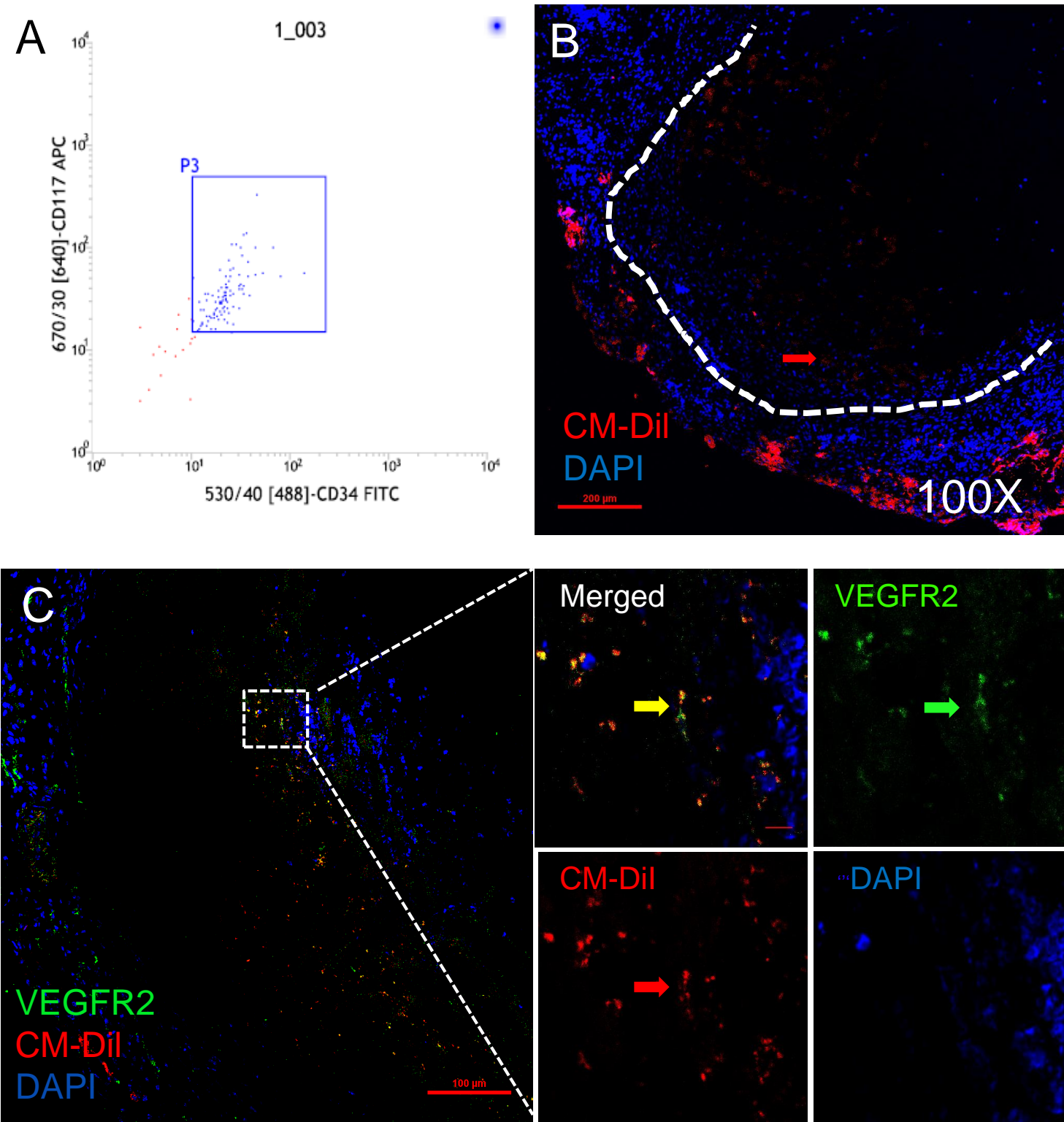

Supplement: Supplementary 3 — Figure III: the migration and differentiation of labeled CD34+CD117+HGSV-AdPC in the thrombus of mice. A The purity of CD34+CD117+HGSV-AdPC detected by FACS (83.5 ± 2.9)% before transplantation. B The CM-Dil-labeled CD34+CD117+HGSV-AdPC enters into the thrombus at posttransplantation day 7. C The result of immunofluorescent staining with antibody VEGFR2. The green arrows indicate VEGFR2+ cells. The red arrows indicate CM-Dil-labeled CD34+CD117+HGSV-AdPC. The yellow arrow indicates transplanted CD34+CD117+HGSV-AdPC differentiation into endothelial cells. Scale bars: 200 μm (B); 100 μm (C); 25 μm (Merged). Magnification: 100x (B, C); 400x (Merged). [file 8816763.f3.pdf]
